# Hypertonicity: Pathophysiologic Concept and Experimental Studies

**DOI:** 10.7759/cureus.596

**Published:** 2016-05-02

**Authors:** Christos Argyropoulos, Helbert Rondon-Berrios, Dominic S Raj, Deepak Malhotra, Emmanuel I Agaba, Mark Rohrscheib, Zeid Khitan, Glen H Murata, Joseph I. Shapiro, Antonios H Tzamaloukas

**Affiliations:** 1 Department of Medicine, Division of Nephrology, University of New Mexico School of Medicine; 2 Department of Medicine, Renal-Electrolyte Division, University of Pittsburgh Medical School; 3 George Washington University; 4 University of Toledo; 5 Department of Medicine, Jos University Teaching Hospital, Jos, Plateau State, Nigeria; 6 Raymond G. Murphy VA Medical Center, Albuquerque, New Mexico; 7 The Joan C Edwards College of Medicine of Marshall University, Huntington, WV; 8 University of New Mexico School of Medicine

**Keywords:** hypertonicity, osmolality, osmolarity, osmolytes, serum sodium concentration

## Abstract

Disturbances in tonicity (effective osmolarity) are the major clinical disorders affecting cell volume. Cell shrinking secondary to hypertonicity causes severe clinical manifestations and even death. Quantitative management of hypertonic disorders is based on formulas computing the volume of hypotonic fluids required to correct a given level of hypertonicity. These formulas have limitations. The major limitation of the predictive formulas is that they represent closed system calculations and have been tested in anuric animals. Consequently, the formulas do not account for ongoing fluid losses during development or treatment of the hypertonic disorders. In addition, early comparisons of serum osmolality changes predicted by these formulas and observed in animals infused with hypertonic solutions clearly demonstrated that hypertonicity creates new intracellular solutes causing rises in serum osmolality higher than those predicted by the formulas. The mechanisms and types of intracellular solutes generated by hypertonicity and the effects of the solutes have been studied extensively in recent times. The solutes accumulated intracellularly in hypertonic states have potentially major adverse effects on the outcomes of treatment of these states. When hypertonicity was produced by the infusion of hypertonic sodium chloride solutions, the predicted and observed changes in serum sodium concentration were equal. This finding justifies the use of the predictive formulas in the management of hypernatremic states.

## Introduction and background

Maintenance of constant volume is of great importance for proper function and survival of body cells [1]. The regulation of cell volume under normal conditions is the consequence of two properties of cells, their ability to extrude solute into the extracellular compartment (EC), and the high permeability of the cellular membranes to water. The main mechanisms of change in cell volume are disturbances in body fluid tonicity (effective osmolality). Tonicity is the property of a solution to make cells suspended in it swell by gaining water (hypotonicity) or shrink by losing water (hypertonicity) through osmotic pressure differences between the intracellular compartment (IC) and the solution tested. Solutions are isotonic when the volume of cells suspended in them does not change by osmotic fluid transfers. Disturbances in tonicity have clinical consequences. The brain is the site of the principal clinical manifestations of both hypotonicity and hypertonicity. The neurological manifestations of either hypotonicity or hypertonicity may lead to irreparable deficits or death. Consequently, proper management of disturbances in tonicity is critical and requires an understanding of the qualitative and quantitative aspects of their pathophysiologic mechanisms.   

The purpose of this review is to explore the evolution of the quantitative and qualitative aspects of the concept of hypertonicity and to summarize the experimental work, which has led to the current understanding of this topic.

## Review

### Physicochemistry 

#### Definitions of Osmosis, Osmotic Pressure, Osmolality, Osmolarity and Tonicity

Water transfers from or into the cells and, therefore, changes in cell volume are secondary to osmotic pressure differences between the IC and the EC. Osmosis is the net movement of water across a selectively permeable (semipermeable) membrane caused by water concentration difference. Cell membranes are highly permeable to water, less permeable to electrolytes, and essentially impermeable to large organic polyanions. In a system of two compartments separated by a semipermeable membrane, osmotic pressure is measured by the hydrostatic pressure that is needed on the side of the higher solute concentration to stop net osmotic water movement across the membrane. Osmotic pressure ($\pi$) is mathematically expressed by van’t Hoff’s law as follows:

                                                                $\pi = C\times R\times T$           {1}

where C is the concentration of solutes in Osm/L, R is the ideal gas constant which is equal to 62.36 L×tor/(mol×K), and T is the absolute temperature in degrees Kelvin (K = 273 + actual temperature in centigrade degrees). If body temperature is considered constant at 37^o^ centigrade, then both T (310^o^ Kelvin) and R (and consequently, the product R × T) are constant and osmotic pressure is a linear function of the osmolar concentration of body fluids. In clinical practice, osmolar concentration is expressed as osmolality (mOsm/kg water in the solution) or osmolarity (mOsm/L of the solution). Osmolality provides an accurate measurement of solute concentration in biological fluids. Osmolarity is less accurate than osmolality because it is affected by the water content of the solution (the fraction of the volume of the solution that is water). Differences between osmolality and osmolarity are encountered in serum measurements. Osmotically active solutes are found only in the water phase of the serum. The average water content of the serum is 0.93 under normal conditions of hydration and serum solids. Therefore, serum osmolality exceeds serum osmolarity by approximately 7% under normal conditions. Serum osmolarity underestimates the true solute concentration in water in clinical states causing an elevation of serum solid content (e.g., hyperproteinemia, hyperlipidemia). In this case, the difference between serum osmolality and osmolarity exceeds 7%. This discrepancy is the cause of diagnostic difficulties.

The relationship between osmotic pressure, expressed in mm Hg, and osmolality, expressed in mOsm/kg, for a solution with an osmolar concentration of 1 Osm/kg can be calculated as follows: $\pi$ = 62.36 × 310 = 19,332 mm Hg, or (at first approximation) $\pi$ = 19.3 mm Hg for each mOsm/kg. A dilute solution of a completely dissociated monovalent salt, such as sodium chloride, will provide two dissolved particles per molecule. Because of interionic attraction forces, ions do not behave as entirely independent particles in biological fluids. Correction for this apparent deviation from van’t Hoff’s law is achieved by using an empirical factor, the osmotic coefficient, which is considered to be equal to 0.93 for monovalent sodium salts in solutions with sodium concentration equal or close to the normal serum sodium concentration. For example, isotonic saline (0.9% or 154 mmol/L sodium chloride in water) with a water content of almost 100% would have a theoretical osmolality of 308 (154 sodium + 154 chloride) mOsm/kg. Its actual osmolality is 0.93 × 308 = 286 mOsm/kg. Serum osmolarity is not measured. It is calculated as the sum of the osmotic concentrations of sodium salts, urea, and glucose in serum volume. The osmotic coefficient of sodium salts is usually taken to be equal to 2.0 in these calculations. The error in the estimate of osmolarity from this calculation is considered negligible. The total concentrations of osmotic substances in plasma, interstitial fluid, and intracellular fluid are equal in the steady state [2]. 

Osmolality and tonicity of a solution, although related, are not the same. The total concentration of dissolved solutes in a solution (the osmolality of this solution) determines its colligative properties (the degree of lowering of the freezing point, elevation of the boiling point, or depression of the vapor pressure from the corresponding value of pure water). Osmolality is measured by instruments calibrated to express changes in a colligative property (usually depression of the freezing point) in units of osmolality (mOsm/kg). The osmolality of a solution represents the sum of the osmotic concentrations (the concentrations times the corresponding osmotic coefficients) of all dissolved solutes. Tonicity of a solution is the part of its total osmolality that can cause steady state osmotic water transfers between IC and EC. The tonicity of a solution is evaluated by the photographic recording of rapid changes in the volume of cells, usually red cells, suspended in this solution. This technology has been applied only in research studies. Clinically, tonicity is estimated as the part of osmolarity attributed to solutes with extracellular distribution (see below).

#### Gibbs-Donnan Equilibrium

The IC contains large molecular weight polyanions (e.g., proteins), to which the cell membranes are, by and large, impermeable. Although its composition varies between tissues and by distance from cell membranes, the fluid in the interstitial compartment, which is the part of EC that is in direct contact with the cells, is considered to have negligible concentrations of polyanions in general. Intracellular polyanions electrostatically attract cations and repel anions across the cell membrane. The Gibbs-Donnan relationships express the equilibrium status of electrolytes in a system of two compartments separated by a semipermeable membrane when one of the compartments contains polyanions. Understanding this equilibrium is required for analyzing water exchanges between the two compartments.

The Gibbs-Donnan equilibrium states that if there are impermeable anions in one of two compartments separated by a semipermeable membrane: (a) the concentration of electrolyte cations is higher and the concentration of electrolyte anions is lower in the compartment with the impermeable anions; (b) the concentration of anions and cations is equal in the compartment that does not contain impermeable anions; and (c) the products of the molar concentrations (anions times cations) are equal in the two compartments. The mathematical expression of the Gibbs-Donnan equilibrium between the EC and the interstitial compartment is as follows:

                                                               $[C]e = [A]e$              {2}

                                                               $[C]i > [A]i$               {3}

                                                  $[C]e\times [A]e = [C]i] \times [A]i$_                            _{4}

where [C]_e_ and [A]_e_ are the extracellular cation and anion electrolyte concentrations and [C]_i_ and [A]_i_ the interstitial cation and anion electrolyte concentrations, respectively.

The Gibbs-Donnan equilibrium predicts that the sum of anions, plus cations, which is by far the major constituent of osmolality of body fluids under normal conditions, is higher in the IC than in the EC. This is a consequence of a geometric rule relevant to the Gibbs-Donnan equilibrium. The rule states that the square has the smallest perimeter of all the rectangles that have the same surface area. For example, a square with a 6 m side and a rectangle with sides of 4 and 9 m have the same surface area (6 × 6 = 4 × 9 m^2^). This relationship would fulfill the requirements of equation 4. However, the perimeter of the square (4 × 6 = 24 m) is less than that of the rectangle (2 × 4 + 2 × 9 = 26 m), and consequently, half of the perimeter of the square (2 x 6 = 12 m), which is an expression of equation 1,  is less than that of the rectangle (4 + 9 = 13 m), which is an expression of equation 3. As a consequence of this geometric rule, the sum of the anions and cations (the osmolality) would be higher in the IC compartment than in the interstitial compartment; consequently, an osmotic pressure difference favoring translocation of interstitial fluid into the IC and cell swelling exists, creating the need for constant removal of solute from the IC in order to maintain a constant cell volume. Maintenance of a constant cell volume requires consumption of energy. Continuous sodium transfer outside the IC under the influence of Na^+^-K^+^ ATPase is the main energy-consuming physiologic mechanism protecting the volume of the cells. Cells swell under anoxic conditions or under the influence of drugs inhibiting Na^+^-K^+^ ATPase [1].

### Response of body cells to hypertonicity

#### Principles Governing the Distribution of Water Between the Major Body Compartments

The fundamental cell properties that are pertinent to the maintenance of cell volume in the normal state and responsible for clinical abnormalities in cell volume during disturbances in tonicity are the high permeability of cell membranes to water, which is universal with the exception of the membranes of the cells in the cornea and, in the absence of vasopressin, of the apical membranes of the principal cells in the renal medullary collecting ducts, and the machinery to continuously expel cations from the cells (the Na^+^-K^+^ ATPase). Cell volume abnormalities develop during disturbances of body water and solute balance and can be quantitated by applying two principles that emanate from the high permeability of cell membranes to water, the osmotic principle and the principle of body water distribution. 

The osmotic principle states that in the steady state osmolality is the same in the IC and EC [3]. Experimental testing of the osmotic principle by measuring osmolality in serum and several body tissues proved to be technically difficult and proof for this principle became available approximately 15 years after Peters stated the principle [4-5].

The principle of body water distribution was formulated first [6], although it is a direct consequence of the osmotic principle. This principle states that, in the steady state, body water is distributed between the EC and the IC in proportion to the amount of solute in each compartment [6]. Quantitative analyses of abnormalities in tonicity are routinely based on the principle of body water distribution [7-14]. The formulas derived from these quantitative analyses guide quantitative treatment prescriptions for clinical states of dystonicity. The derivation, experimental testing, and limitations of these formulas should, therefore, be understood.

#### General Mechanisms of Hypertonicity

Rises in tonicity result from changes in body water, body solute, or both water and solute. Combined gains or losses in water and solute generated through interactions between the EC and the external environment are the more common sources of hypertonic abnormalities. The loss in body water alone is seen in some clinical states (for example, in diabetes insipidus). Hypertonicity caused by net solute gain without a change in body water is uncommon. An example of this last hypertonic condition is hyperglycemia resulting from excessive hepatic glucose production during insulin deficiency states occurring in anuric subjects. 

In terms of their ability to cause disturbances in tonicity, solutes are classified into two categories, those that do and those that do not cause steady state changes in cell volume. Increases in the body content of endogenous (e.g., urea) or exogenous (e.g., alcohols) solutes distributed in total body water cause rises in body fluid osmolality but do not cause changes in cell volume in the steady state. The clinical manifestations and treatment of excesses in the body content of solutes with body water distribution are different from those of hypertonicity [15-18]. Increases in the body content of solutes with EC distribution, whether endogenous (glucose) or exogenous (salt, mannitol), cause hypertonicity [19-20]. If the solute causing hypertonicity is not a sodium salt, the exit of water from the IC in hypertonic states results in dilution of the EC sodium salts, which in the normal state are the main EC solutes [19]. Tonicity is estimated as the sum of serum sodium concentration times its osmotic coefficient, plus the osmotic concentration of glucose and/or any other solute with extracellular distribution.

Most solute abnormalities leading to tonicity disturbances are accompanied by changes in body water. Not taking into account the change in body water, regardless of how small it is, in the calculations of fluid shifts between the IC and EC and of osmolality changes in disturbances of tonicity can be the source of disproportionately large errors [8].

#### Mathematical Models of Hypertonicity

Traditionally, models of hypertonicity predicted the degree of increase in tonicity, gain in EC volume, and loss in IC volume after a hypertonic disturbance of known volume and tonicity by assuming that cells behave as perfect osmometers. Other gains or losses of water and solute, principally through the urine, routinely interfere with the magnitude of the disturbances in tonicity in clinical practice. These other gains or losses were not accounted for in the predictive models [7-14]. Therefore, the existing mathematical models of hypertonicity are strictly applicable only to anuric states.

**Figure 1:**
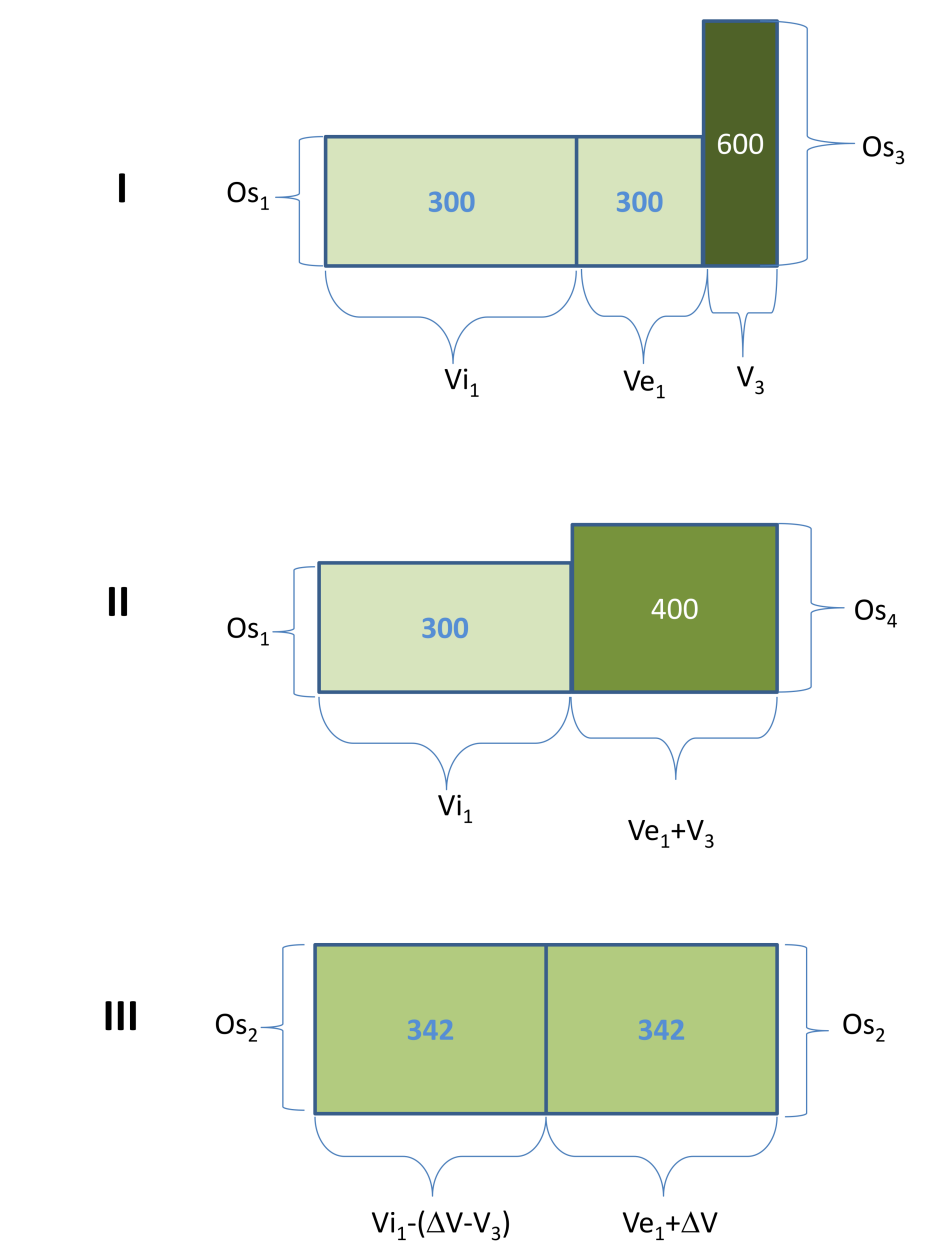
Stages of hypertonicity caused by extracellular gain of a hypertonic solution. Stage I: Addition of a hypertonic solution to the EC prior to any mixing between the EC and the hypertonic solution. Stage II: Hypothetical stage of complete mixing of EC fluid and infused hypertonic solution prior to any interaction with the IC. Stage IIII: Final steady state after osmotic transfer of water from the IC into the EC that led to equal osmolalities in the two compartments. Ordinates: volumes. Abscissae: osmolalities. The figure was constructed assuming that V_i1_ = 2xV_e1_, V_3_ = 0.5xV_e1_, and Os_3_ = 2xOs_1_. The numbers within the boxes show osmolalities.

Figure 1 shows the determinants of the changes in tonicity, body water, and body water distribution in hypertonic states secondary to extracellular gains of hypertonic solutions in anuria [11]. Table 1 explains the symbols for osmolalities and volumes in Figure 1.

**Table 1:** Symbols Used in Figure 1.

Ordinates Osmolalities (Os)	Abscissae Volumes (V, $\Delta$V)	Ordinates Times Abscissae Solute (mOsm)
Os_1_ = baseline	V_e1_ = baseline EC	Os_1 _x V_e1_ = baseline EC
Os_2_ = final	V_i1_ = baseline IC	Os_1 _x V_i1_= baseline IC
Os_3_ = infusate	V_1_ = V_e1_ + V_i1_ = baseline body water	Os_3 _x V_3_ = infused
Os_4_ = Hypothetical EC osmolality after equilibration of osmolality in the EC but before any interactions between EC and IC	V_3_ = infusate (added) \$\Delta$V = final EC gain	Os_4 _x (V_e1_ + V_3_) = final EC
	$\Delta$V +V_e1_ = final EC	Os_2 _x (V_e1_ + $\Delta$V) = final EC, Note: Os_4 _x (V_e1_ + V_3_) = Os_2_x(V_e1_ + $\Delta$V)
	$\Delta$V – V_3_ = osmotic volume transfer from IC to EC	[Os]_2 _x (V_i1_ – [$\Delta$V – V_3_]) = final IC, Note: [Os]_2 _x (V_i1_ – [$\Delta$V – V_3_]) = Os_1_xV_i1_
	V_i1_ – ($\Delta$V – V_3_) = final IC	

The necessary and sufficient conditions for cells to be considered as perfect osmometers are that total EC solute remains constant between stages II and III, total IC solute remains constant between stages I, II, and III in Figure 1, and that equilibration of osmolalities between the IC and EC is achieved exclusively by water transfer from the IC into the EC. Under these conditions, the volumes V_i1 _(baseline IC volume), V_e1_ (baseline EC volume), V_1_ (baseline body water or V_i1_ + V_e1_), and V_3_ (volume of the infusate) and the osmolalities Os_1_ (baseline osmolality) and Os_3_ (osmolality of the infusate) are the independent variables, while the volume $\Delta$V (the difference between the final and baseline EC volumes) and the final osmolality (Os_2_) are important dependent variables that can be calculated from the independent variables by the following formulas [11].

Total EC volume expansion (\(\Delta\V) = osmotic shift of water from the IC into the EC, plus volume infused):

                      $\Delta V = V3\times (Os1\times Ve1 + Os3\times [V3 +Vi1])/(Os3\times V3 +Os1\times V1)$                     {5}

For expansion with water (Os_3 _= 0), equation 5 becomes:

                                          $\Delta V = V3\times Ve1/V1$                {6}

For isotonic expansion (Os_3_ = Os_1_ = Os_2_), equation 5 becomes:

                                            $\Delta V = V3$                       {7}

Final osmolality:

                                          $Os2 = (Os1\times V1 + Os3\times V3)/(V1 + V3)$                     {8}

If the infusate is hypertonic saline, equation 8 can be modified to calculate the final EC sodium concentration as follows:

                                    $[Na]2 = ([Na]1\times V1 + [Na]3\times V3)/(V1 + V3)$                     {9}

where [Na]_3_ = sodium concentration of the infusate, [Na]_1_ = initial EC sodium concentration, and [Na]_2_ = final EC sodium concentration (note: although rapidly exchangeable sodium is distributed, by and large, in the EC, the changes in EC sodium concentration during disturbances in tonicity are defined by total body water. This is a consequence of the osmotic principle). Whether the large amount of sodium that is bound to polyanionic proteoglycans primarily in cartilage, bone, and skin is available for rapid participation in tonicity disturbances is not clear [21-22]. 

Formula 9 uses the same mathematical determinants of the change in serum sodium concentration as the Adrogue-Madias formula, which is currently used to calculate the increase in serum sodium concentration ([Na]_2_ – [Na]_1_) after infusion of one liter of a saline solution with a higher sodium concentration than the serum sodium concentration and is expressed by the following relationship [10]:

                               $[Na]2 - [Na]1 = ([Na]3 - [Na]1])/(V1 + 1)$                                   [10]

Formulas 9 and 10 provide essentially the same results [12].

If final osmolality (Os_2_) is known, the initial fluid volume that determines the final osmolality (V_Osm_) after infusion of an amount of solute equal to Os_3_×V_3_ is calculated by a modification of the Fick equation that takes into account the volume infused as follows [11]:

                             $VOsm = V3\times(Os3 - Os2)/(Os2 - Os1)$                 {11}

Formula 11 calculates the so-called “osmotic volume of distribution”, which, under perfect osmometer conditions, should be equal to the baseline body water (V_1_) [7, 9]. If the EC expansion is done with hypertonic saline, equation 10 can be modified to calculate the sodium volume of distribution, as follows:

                           $VNa = V3\times([Na]3 - [Na]2)/([Na]2 - [Na]1)$             {12}

Under conditions of a perfect osmometer, V_Na_, V_Osm_, and V_1_ should be equal [7, 11]. Thus, equations 11 and 12 allow the experimental estimation of body water. Comparison of body water estimates obtained from equation 11 and simultaneous body water measurements obtained by standard methods, such as isotopic water dilution, allowed testing of the hypothesis that cells behave as perfect osmometers [7-9].

#### Experimental Testing of the Perfect Osmometer Hypothesis

**Figure 2:**
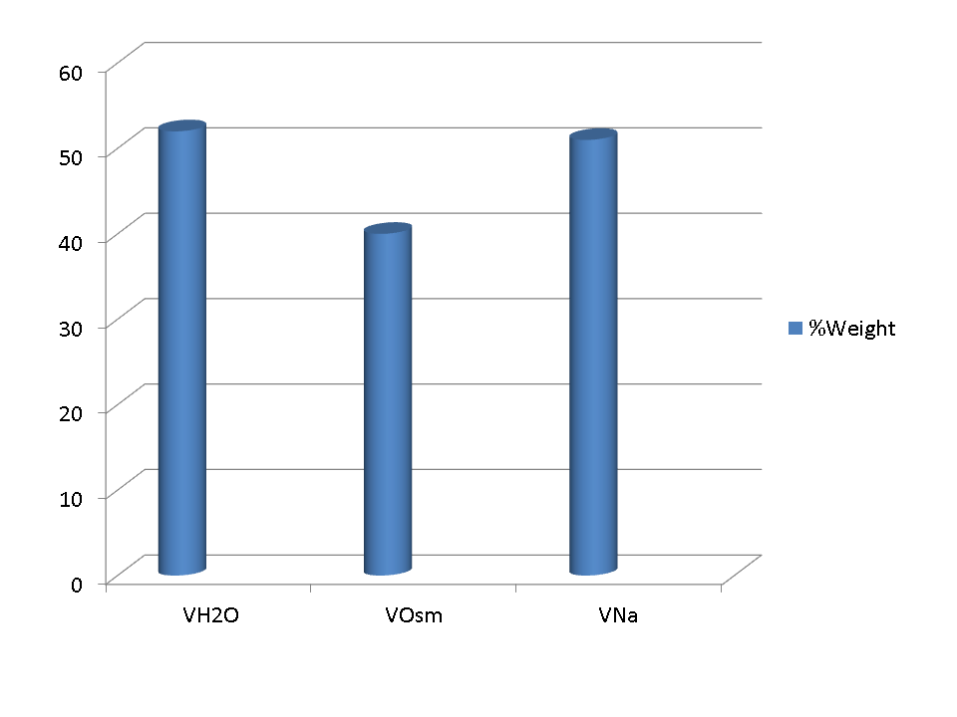
Estimates of body water and the osmotic and sodium volume of distribution in acute hypernatremia. Shown here are the average estimates of body water (VH_2_O) measured by tritiated water dilution, the osmotic volume of distribution (VOsm) calculated by formula 11, and sodium volume of distribution (VNa) calculated by formula 12 in a study of anuric dogs infused with hypertonic saline [26]. The figure makes two points: (a) Even in acute hypertonicity, VOsm is substantially lower than VH_2_O and (b) VNa is equal to VH_2_O. The equality of VNa and VH_2_O provides the basis for applying equations 9 or 10 in the management of dysnatremias.

Because the mathematical models of the perfect osmometer hypothesis ignored the renal contribution to body fluid balance, experimental testing of its models and of metabolic abnormalities that result from hypertonicity, such as acid-base disturbances and hyperkalemia, has usually been carried out by infusion of hypertonic solutions into anephric or anuric animals [7-8, 23-26]. In addition to hypertonicity, these experiments resulted in EC expansion by the volume infused and the volume osmotically transferred from the IC. The results of these early experiments are summarized below: 

(a) The volume of the infused solution (V_3_) has substantial effects on the results and must be accounted for, even when it is small as it is in hypertonic infusions [8]. Equations 5-12 represent mathematically rigorous expressions of the perfect osmometer hypothesis because they include all the determinants of changes in osmolality and body water distribution, including the volume V_3_ [9, 12]. 

(b) In hypertonic saline expansion, the final serum sodium concentration is predicted accurately by equation 9 and V_Na_ estimated from equation 12 is equal to total body water determined by reference methods. However, final osmolality is higher than the value predicted by equation 8 and, consequently, V_Osm_ is less than either body water or V_Na_ [8, 26]. This finding led to the conclusion that hypertonic expansion produces new solute, the so-called “idiogenic osmoles” [8]. Hypotonic expansion experiments with water confirmed the finding that V_Na_ is equal to body water [27-28]. 

(c) Experimental testing of the perfect osmometer hypothesis was traditionally done by infusing hypertonic solutions into acutely anuric or anephric experimental animals [7-8, 26]. In addition to hypertonicity, these experiments caused substantial extracellular expansion and progressive azotemia, conditions leading to the generation of new solutes, not as a direct consequence of hypertonicity. Extracellular expansion dilutes certain solutes, for example, glucose and calcium, the extracellular concentration of which is tightly regulated by hormones. Rapid responses of the regulatory hormones cause the release of these solutes in the extracellular compartment from stores that were not osmotically active previously [26]. Similarly, progressive azotemia indicates the progressive generation of new solute, primarily urea. Correction for the new solute generated by extracellular expansion and azotemia accounted for only part of the “idiogenic osmoles” [10]. Figure 2 shows the estimates of body water, V_Na_ , and V_Osm_ calculated after infusion of hypertonic saline in one experimental study [26].

Although estimates of V_Na_ and body water are remarkably similar under a variety of experimental conditions, the discordance between V_Osm_ and body water mandated the study of “idiogenic osmoles”, which have, after their identification, been renamed “organic osmolytes”.

#### The Nature of “Idiogenic Osmoles”

The brain was identified early as an important site of deviation from perfect osmometric behavior in acute experimental hypertonicity. Hypertonic states reduce brain volume. However, even in acute hypertonic states, the reduction in brain volume is substantially less than the reduction predicted from calculations based on perfect osmometric behavior [29].

Astrocytes play a major role in the defense of brain volume in acute states of dystonicity [30]. Immediate adaptation to brain shrinking includes movement of fluid from the cerebrospinal fluid into the astrocytes. This is a limited adaptive mechanism. The main adaptation occurs by a process called regulatory volume increase (RV), which consists of intracellular solute gain.

**Figure 3:**
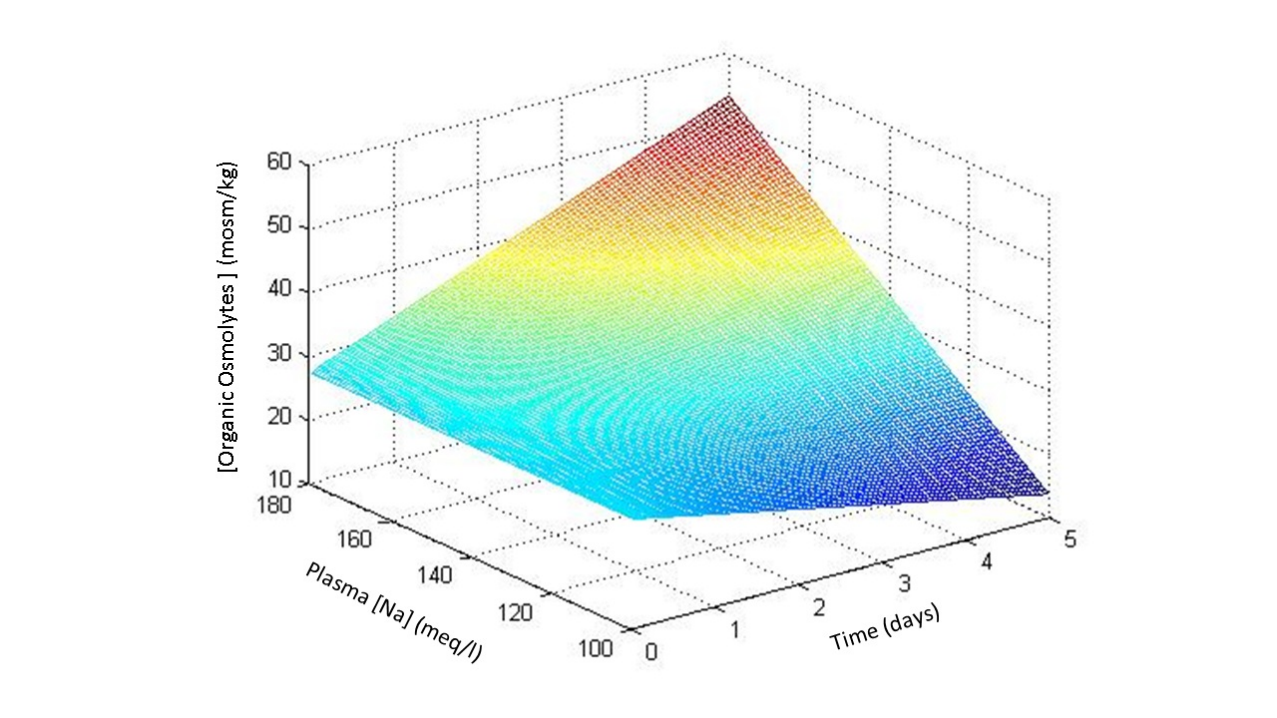
Time course of brain organic osmolytes in hypertonicity. From experimental studies in rats subjected to acute and chronic hypernatremia [35] and from the stage of correction of experimental chronic hyponatremia [36].

The early component of RV in hypertonic states is accomplished within 30-120 minutes of the hypertonic stimulus by the rapid influx of ions (sodium, chloride, and potassium) from the interstitial compartment (which shrinks) into the brain IC [31-33]. In chronic hypertonicity, which develops in days or weeks rather than minutes or hours, brain volume is restored by the accumulation of organic compounds, the so-called “brain osmolytes” [34]. Figure 3 shows the sequence of gains in brain osmolytes in acute and chronic hypertonicity [35-36].

A number of major and minor organic osmolytes has been identified in the brain [34]. Glutamate, glutamine, taurine, and myoinositol are considered as major osmolytes while aspartate, alanine, glycine, choline, lysine, serine, threonine, glycerophosphorylcholine (GPC), betaine, gamma-aminobutyric acid (GABA), and phosphocreatine are classified as minor osmolytes [34]. Some of these osmolytes are amino acids acquired from the circulation while others are polyols (myoinositol) or methylamines (betaine, GPC) formed locally. At least one osmolyte (GABA) is a neurotransmitter. Organic osmolyte acquisition accounts for 30-50% of the solute accumulation in the brain IC in chronic hypertonicity [34-35, 37-43] and is slow, requiring two days or more to reach a steady state. Renal medulla is physiologically subjected to rapid variations in interstitial tonicity and is endowed with a rapid mechanism of modifying its intracellular osmolyte content. The main osmolytes in the renal medulla are sorbitol, myoinositol, GPC, and betaine [44].

Water administration in a state of acute hypertonicity leads to rapid loss of the excess electrolytes and rapid restoration of the brain cell volume to normal. After correction of chronic hypertonic states by water administration, organic osmolyte brain content declines over two days [35].

#### Mechanisms of Intracellular Solute Gain in Hypertonicity

Hypertonicity causes activation of several ion transporters in cell membranes. Activation of the Na^+^, K^+^, 2Cl^-^ cotransporter leads to the intracellular transfer of sodium chloride and potassium chloride. Tandem activation of Na^+^/H^+^ exchange and Cl^-^/HCO_3_^-^ exchange leads also to sodium chloride entry into the cells. Sodium ion entering the cells is extruded through Na^+^/K^+^ ATPase in exchange for potassium. Potassium chloride is the final salt gained intracellularly in hypertonicity [45].  

The mechanisms responsible for the intracellular accumulation of organic osmolytes in hypertonic states include intracellular formation and transport. Examples of solutes acquired by intracellular formation include sorbitol formed from glucose under the catalytic action of activated aldose reductase, GPC formed from phosphatidylcholine under the influence of phospholipase A_2_, and amino acids derived from autophagic proteolysis. Examples of organic osmolytes acquired by intracellular transport include myoinositol, betaine, and taurine, which are acquired through the action of Na^+^ coupled transporters [45]. Hypertonicity activates a variety of genes regulating metabolic functions and transporters responsible for the intracellular accumulation of organic osmolytes [46]. Tonicity enhancer binding protein (TonEBP) is a transcription factor found in abundance in the brain, renal medulla, heart, liver, and activated T cells that plays a major role in hypertonicity and immune phenomena [47]. This factor is activated by hypertonicity and regulates the expression of several osmolyte transporters [47-48].

The alterations of metabolism induced by hypertonicity have dire consequences. Severe hypertonicity leads to cell death by apoptosis. Apoptotic pathways activated by hypertonicity include activation of caspase-8 and release of cytochrome 3 with activation of caspase-3 and caspase-9 [49]. Elevated levels of the tumor suppressor p53, which initiates apoptotic pathways in hypertonicity, is an important apoptotic mechanism activated by hypertonicity but apparently not the only one [50].      

## Conclusions

Formulas used to calculate the volume of hypotonic solutions needed to correct the hypertonic states do not take into account ongoing fluid losses during treatment, which should be replaced. The same formulas also fail to account for the intracellular acquisition of new solute created by the hypertonicity. This new solute is the source of serum osmolality values substantially higher than those predicted by the formulas and may cause severe complications during correction of the hypertonic states. When the hypertonic states are secondary to conditions of known magnitude causing hypernatremia, the degree of observed elevation in the serum sodium concentration is equal to the degree predicted by the formulas. This last observation justifies the application of predictive formulas in the treatment of hypernatremia, provided ongoing fluid losses are also addressed.
